# The WALLET Study: Examining Early Memory Loss and Personal Finance

**DOI:** 10.1093/geroni/igac038

**Published:** 2022-05-26

**Authors:** Peter A Lichtenberg, Wassim Tarraf, Vanessa O Rorai, Matthew Roling, Juno Moray, Evan Z Gross, Patricia A Boyle

**Affiliations:** Institute of Gerontology, Wayne State University, Detroit, Michigan, USA; Institute of Gerontology, Wayne State University, Detroit, Michigan, USA; Department of Healthcare Sciences, Wayne State University, Detroit, Michigan, USA; Institute of Gerontology, Wayne State University, Detroit, Michigan, USA; School of Business, Wayne State University, Detroit, Michigan, USA; Institute of Gerontology, Wayne State University, Detroit, Michigan, USA; Rehabilitation Institute of Michigan, Detroit, Michigan, USA; Rush Alzheimer Disease Center, Rush University Medical Center, Chicago, Illinois, USA

**Keywords:** Financial decision-making, Financial exploitation, Financial management

## Abstract

**Background and Objectives:**

This feasibility study tests a new approach for assessing personal finance in older persons with early memory loss. The project examines 2 primary outcomes that gauge the financial viability and well-being of older adults: wealth loss and financial exploitation. The overall objective is to determine the association of financial literacy and management, financial decision-making, and cognition with wealth loss and financial exploitation.

**Research Design and Methods:**

This cross-sectional study recruited 46 participants who were 60 years of age or older. Participants were classified as having mild cognitive impairment, perceived cognitive impairment, or no cognitive impairment. The study coordinator arranged with each participant to obtain copies of their main checking account statements for 12 consecutive months within the previous 2 years and, if appropriate, credit card statements. All statements were de-identified and assigned a random ID number. Participants then completed 2 telephone interviews.

**Results:**

The average participant age was 72 years (standard deviation [*SD*] = 7.7); 84% were female, 39% White, and 35% currently married. Average education was 16.2 years (*SD* = 2.4); mean yearly household income was almost $42,000 (*SD* = 25,752); and monthly social security payments averaged $1,446 (*SD* = 1,244). Our results indicate that the methods used to analyze checking account statements, followed by telephone interviews to verify identified trends, were useful in developing a financial behavior index to measure wealth loss.

**Discussion and Implications:**

We demonstrate an alternative method for assessing personal finance using person-centered principles, which we believe are critical in the presence of diminished or impaired cognition. Our findings offer an innovative method for assessing the risk for wealth loss and financial exploitation.

**Translational Significance:** Our study offered researchers and clinicians a new way to assess personal finance. We demonstrated that a person-centered approach to analyzing personal finance is feasible and may uncover specific financial behaviors that put older persons at risk for wealth loss and exploitation. Specifically, we found that we could systematically review personal checking account statements and, through follow-up interviews, confirm, and/or expand on important variables, such as annual income, expenditures per category, late fees, or missed bills and offer consistent financial help to others.

In 2016, the Institute of Medicine and the National Academy of Sciences sponsored a committee to evaluate the Social Security Administration’s capability determination process for adult beneficiaries. In their final report, the committee recommended that financial capacity should be defined by and assessed as real-world performance in meeting one’s basic needs and success in handling financial demands in the individual’s actual environment. Although recent research has underscored the need for such an approach, no one has proposed a real-world approach to daily personal financial management. As described later in the literature review, a person-centered approach to financial decision-making has proved reliable and valid. Extending these person-centered principles to financial management may prove fruitful. If this is the case, a novel method for assessing financial management skills may result. The measurement of real-world financial management, as alluded to by the Institute of Medicine and National Academy of Science (IOM/NAS), can enhance the assessment of financial capacity skills and improve the ability to detect declines in specific domains of financial capacity before a crisis occurs in an individual’s personal finances. This article describes the feasibility of a novel measurement approach to the assessment of real-world personal finance and wealth loss for older persons with and without early memory loss: the Wealth Accumulation and Losses in Late life Early Cognitive Transitions (WALLET) study.

## The Domains of Financial Capacity

Marson (2001, 2016) reviewed approaches to understanding financial capacity in older adults and such studies’ research findings. Essentially, the domains of financial capacity for personal finance are intact financial management, financial decision-making, and the ability to avoid financial exploitation (FE). Marson (2001) created a financial competency inventory (FCI) that uses neutral stimuli to assess the domains of financial capacity. We know from the extensive work of Marson et al. that financial capacity declines in persons with mild cognitive impairment and Alzheimer’s disease, as assessed by the FCI.

In a separate set of studies, other researchers used neutral financial decision-making stimuli and found links between decreased cognition and decreased financial decision-making, which is one of the domains of financial capacity. In a sample of over 400 older adults, Boyle et al. (2012) found that even subtle age-related cognitive decline (i.e., decline that would not be in the range of cognitive impairment) was related to lower financial decision-making. Furthermore, Stewart et al. (2019) found that older persons without cognitive impairment but with decision-making deficits were more than twice as likely to develop incident dementia. Financial decision-making may well be a related but separate construct from cognition. Researchers in the Rush University Memory and Aging Project (Boyle et al., 2012; Han et al., 2015) examined the relationship between cognition and financial decision-making longitudinally. Importantly, findings from the Rush group suggest that decision-making and cognition are related but relatively distinct constructs, and that decision-making is highly influenced by psychological factors (Han et al., 2015). These two research programs yielded two major findings: (1) that financial capacity measured through neutral stimuli is affected by cognitive decline and dementia and (2) both financial management and financial decision-making skills are related to declining cognition.

## Financial Capacity and Wealth Loss in Early Cognitive Impairment

Recent research using the health and retirement study (HRS) highlights the risk of wealth loss during early cognitive impairment. Using the HRS, Hsu and Willis (2013) found that declines in financial management skills (e.g., paying bills) were largely related to an older person’s cognitive skills. Angrisani and Lee (2018) examined the relationship between cognitive loss and private wealth loss using HRS data and found that significant memory loss across a 4-year period was associated with an average reduction in wealth in the memory-impaired group that was more $30,000 more than the loss in the nonimpaired group. Using Medicare claims data across a 19-year period, Nicholas et al. (2021) reported that subprime credit scores and missed bill payments increased significantly shortly after a diagnosis of Alzheimer’s disease. These wealth loss studies describe the association between significant memory loss and wealth loss, as well as the risk for changes in subprime credit scores. The studies did not examine the daily management of finances, and thus could not identify how financial decision-making, financial management behaviors, and FE affect this wealth loss.

## A Person-centered Approach to Financial Decision-making

Lichtenberg et al. (2015) proposed a new conceptual model to understand financial decision-making and for use in the assessment of financial capacity: the Lichtenberg Financial Decision-making Rating Scale (LFDRS). The conceptual frameworks used in creating the LFDRS were the Whole Person Dementia Assessment model (Mast, 2011) and the decision-making model of Appelbaum and Grisso (1988), which elaborates on what Lichtenberg et al. term the intellectual factors involved in capacity assessment: choice, understanding, appreciation, and reasoning. The Whole Person Assessment model is described in some depth in Lichtenberg et al. (2015) and applies person-centered principles of deep respect for individuality and personhood to the standardized psychological assessment process. A central feature is the focus on actual decisions instead of hypothetical vignettes.

The LFDRS incorporates contextual variables (i.e., financial situational awareness [FSA], psychological vulnerability [PV], and susceptibility to undue influence and FE) into Appelbaum and Grisso (1988) decision-making model. These intellectual factors have been established as fundamental aspects of decisional abilities. Although articulated originally for medical decision-making, the same intellectual factors apply to financial decisions. First, the older adult must be able to clearly communicate his or her choice. Understanding is the ability to comprehend the nature of the proposed decision and provide some explanation or demonstrate awareness of its risks and benefits. Appreciation refers to the situation and its consequences, and often involves their affect on both the older adult and others. Appelbaum and Grisso contend that the most common causes of impairment in appreciation are the lack of awareness of deficits and/or delusions or distortions. Reasoning includes the ability to compare options—for instance, treatment alternatives in the case of health care—and provide a rationale for the decision or explain the communicated choice.

The scale developed aims to quantify financial decision-making risk—that is, the risk for meeting the legal standards for financial incapacity and risk for vulnerability to FE. The contextual factors for the RS are FSA; PV, which includes loneliness and depression; and susceptibility to undue influence; and to FE (susceptibility). These contextual factors directly influence the intellectual factors associated with decisional abilities for a significant financial transaction or decision. This financial decision-making rating scale has been linked to both cognitive decline and risk for FE (see Lichtenberg et al., 2016, 2017; Campbell, Gross & Lichtenberg, 2019, for further details). The use of a person-centered approach to financial decision-making is novel, and the results support the use of such an approach. While the scale can assess the quality of financial decision-making, however, it does not address any items related to financial management.

## Purpose of the Study

This feasibility study was designed to examine a person-centered approach to assessment of the daily financial management aspects of personal finance in older persons with early memory loss. Specifically, we examined 12 months’ worth of personal checking account statements from those with early memory loss to identify certain financial behaviors, then used an interview technique to better specify what certain expenditures were for and, in the process, identify predictors of wealth loss and FE. We also explored the relationship of person-centered financial decision-making to financial management and to wealth loss and FE.

## Method

### Procedure for Recruitment

Individuals aged 60 years or older who were primarily responsible for a personal checking account and were English speakers were eligible for this study. Participants were recruited from research registries through the Michigan Alzheimer’s Disease Research Center (MADRC; *n* = 21) and the Wayne State University Institute of Gerontology Healthier Black Elders Center (*n* = 14). Participants were also recruited via newsletters or informational lectures given by the first author. Prospective participants were prescreened to determine eligibility based on the following criteria: age 60 or older and no diagnosis within the last 2 years of epilepsy, stroke, traumatic brain injury, bipolar disorder, or schizophrenia, and no significant use of drugs or alcohol. Participants were then categorized based on their cognitive status. Throughout their longitudinal study, the Michigan Alzheimer’s Disease Research Center used a consensus diagnosis conference process, and the nationally agreed on procedures and definitions for diagnosing mild cognitive impairment. MADRC clinicians categorized participants who complained of memory problems but did not have any deficits in cognitive testing, as having perceived cognitive impairment (PCI). Mild cognitive impairment was diagnosed for other participants only if they had gone through a geriatric memory work-up and received this diagnosis. Other participants were asked whether their memory or problem-solving skills were worse than a year ago. Those who answered yes were categorized as PCI and those who answered no as no memory decline.

The study coordinator arranged with each participant to obtain copies of their main checking account statements for 12 consecutive months within the previous 2 years and, if appropriate, credit card statements. Hard copies were either mailed or hand-delivered, and electronic copies were e-mailed. All statements were de-identified and assigned a random ID number. Participants then completed two telephone interviews. All participants were compensated for their participation and reimbursed for study-associated banking or mailing fees, if any. The study was approved by the Wayne State University IRB.

### Procedure for Analyzing Checking Account Statements and Determining a Financial Behaviors Index

One of our authors (M. Roling), who is an expert in personal finance, led the team in establishing the following procedures for analyzing checking statements and developing the types of questions to ask during interviews. The procedures later are used to identify financial behaviors. These procedures integrated information obtained through our preliminary and in-depth financial interviews as well as through expenses and income streams identified in the check statements.

1. Establish that the participant is the primary manager of the checking account.
2. Establish regular monthly/annual income; this may include multiple sources of income, such as social security, pensions, IRAs with a required minimum distribution, annuities, or other investment accounts. Also, establish whether there are regular payments into a savings or investment account so that these will not be counted as expenditures. Some of these income sources are easily identifiable; others must be probed and confirmed during the interview with the participant.
3. Document and/or calculate monthly inflows and outflows to the checking account. Outflows will be used to determine annual expenditures, and thus probing all such transactions during the interview is crucial.
4. Examine statements for the following: (a) bank fees for insufficient funds, loan interest penalties for missed or late payments, and exorbitant ATM fees and (b) patterns and amounts of cash withdrawals.
5. Examine for one-time, nonrecurring events (i.e., unusual). These include annual tax payments, large purchases, etc. Note any unusual large expenses and clarify what these are during the interview.
6. “Stress test” expense categories are as follows: (a) utility payments—phone, television/computer, water, gas, electricity, etc.; (b) credit card expenses—note amounts, and particularly whether monthly card expenses are the same each month, which likely indicates paying down credit card debt. Then probe for any late payments or penalties. (c) large checks—rent/mortgage, other? (d) multiple payments for the same category; these include insurance, phones, utilities, etc.
7. Reviewing accounts: Determine during the interview how often the older person reviews their bank statements and whether they are aware of how their expenditures match their regular income.
8. Administration and custody: During the interview, determine who has authority/decision-making rights over the account(s); who is available to assist if there’s evidence of possible FE (however, the final determination of FE is the result of a consensus conference with a psychologist and social worker); verify whether the older person is helping others financially on a regular basis.

#### Measures

For our feasibility study, we limited our measures as described later.

##### Wealth loss.―

Wealth loss will be identified through the examination of bank records and during the follow-up interview with the older adult (see procedure for analyzing checking account statements previously). Through these interviews, we will establish the older adult’s annual fixed income (e.g., social security, retirement, and investment returns). The primary determination of wealth loss will be set by subtracting the sum of the 12-month expenditures (from the checking account) from the total fixed income. Negative values will be considered to be wealth loss. For individuals who satisfy the wealth loss criteria, we will translate the loss value to the annual percentage of loss beyond income by dividing the loss value by the annual income. For example, if a participant were to expend $10,000 beyond an income base of $100,000, the loss would be 10%. In follow-up interviews, we will assess the accuracy of our accounting for all income and gauge the participant’s awareness of their wealth loss.

##### Financial exploitation.―

We probe for evidence of this using a variety of methods. One is to probe for unusual payments. A second is through the questions on our financial decision rating scale, which explicitly asks about FE/identity theft/scams. During the interview with the older adult, instances of suspected FE may arise (e.g., as the result of a lottery scam, romance scam, inheritance scam, abuse of trust, and/or financial entitlement leading to the misuse or theft of the older adult’s funds). The WALLET team documents these facts and employs a consensus method of diagnosis by team members (a personal finance expert, a social worker, and the PI, who is a clinical psychologist) to determine whether FE occurred. We were not able to reliably ascertain the exact amounts of money lost.

##### Financial behaviors index.―

We developed this index based on the checking account review and interview of the older person by a simple count of the following: missed bills, late fees, regular financial assistance to others, and overpayment in one expenditure category.

##### Socioeconomic and demographic characteristics.―

Several aspects of participants’ characteristics were captured through survey instruments designed to collect data on demographic, socioeconomic, and physical and mental health factors; cognitive status; and memory functioning. The demographic factors are age, based on birthdate provided by the participant; self-reported gender; race (e.g., White, Black, and mixed race); and marital status. Second, the socioeconomic variables are education, based on the highest level of education completed, annual social security payments, and total household income.

##### Financial decision-making capacity.―

To assess financial decision-making capacity, we used the Lichtenberg Financial Decision Rating Scale (LFDRS; Lichtenberg et al., 2015). This is a clinician-administered scale used to assess financial decision-making ability. The scale contains 56 items across four subscales: (1) FSA, (2) PV, (3) intellectual factors, and (4) susceptibility to undue influence and FE. Interrater reliability and factor analysis that confirm the conceptual model have been documented in previous samples (see Lichtenberg et al., 2015, 2017), as have concurrent validity with cognition (Lichtenberg et al., 2017) and with FE (Lichtenberg et al., 2020). Higher scores reflect more vulnerability across the different factors (i.e., contextual and intellectual) in financial decision-making.

Instrumental activities of daily living (IADL) are the functional abilities entailed in common tasks, such as cooking, transportation, medication, and financial management. The self-report version of the Lawton IADL Scale was administered to participants (Graf, 2008). Based on the participant’s responses to questions, with follow-up for clarification, the examiner rated each participant in eight functional domains on the following scale: 1 = unable, 2 = needs assistance, 3 = some assistance, and 4 = independent.

Finally, the Rey Auditory Verbal Learning Test (RAVLT; Rey, 1958) was used to assess the ability to learn a list of 15 words over five trials. Lezak (1995) described the extensive testing and positive results for the reliability and validity of the RAVLT. Because of coronavirus disease 2019 (COVID-19) restrictions, almost all of our participants were administered this scale orally. We decided to use the raw score on one index from the RAVLT, the Learning over Trials (LoT) test.

##### Analytic approach.―

To gauge the feasibility of the proposed design, we conducted our analyses on a subsample of individuals (*n* = 46) who had completed their interviews and whose data across the WALLET study modules had been generated, quality controlled, cleaned, and processed into usable analytical files. Our analyses proceeded in four steps. First, descriptive statistics were generated to demographically characterize the sample overall and by cognitive impairment status. Second, bivariate correlation analyses were calculated. Third, we provide visual presentations of the distributions of financial behaviors and decision-making indicators by normal cognition and PCI/MCI status. Finally, we estimate penalized maximum likelihood logistic regression models to accommodate the small sample size and a robust linear regression model to estimate incrementally adjusted associations between financial behaviors and financial decision-making and (a) FE and (b) wealth loss, respectively.

##### Preliminary sample description.―

Participant demographics and other measures can be found in Table 1. Forty-six participants were recruited during an 11-month period. Of the 62 older adults who expressed interest in the study and met the criteria, 46 (74%) followed through and completed the study. The average age was 72 years (standard deviation [*SD*] = 7.7); 84% were female, 39% White, and 35% currently married. Average education was 16.2 years (*SD* = 2.4); mean yearly household income was nearly $42,000 (*SD* = 25,752); and monthly social security payments had a mean of $1,446 (*SD* = 1,244). Finally, the average RAVLT-LoT score was 17.28 (*SD* = 8.55), and the mean LFDRS-total score was 13.85 (*SD* = 9.70). These characteristics were consistent across MCI/PCI and cognitively normal (CN) groups as there were no significant differences in memory scores across the groups.

**Table 1.** Sample Characteristics

Variable	Overall (*n* = 46)		MCI/PCI (*n* = 34)		CN (*n* = 12)		*p* Value	SMD
	Mean (*SD*)	*n*(%)	Mean (*SD*)	*n*(%)	Mean (*SD*)	*n*(%)		
Age, years	72.09 (7.69)		71.08 (8.05)		72.45 (7.64)		.602	0.175
Males		7 (15.6)		3 (25.0)		4 (12.1)	.556	0.336
White		18 (39.1)		3 (25.0)		15 (44.1)	.411	0.41
Marital status							.205	0.756
Divorced		11 (23.9)		2 (16.7)		9 (26.5)		
Married		16 (34.8)		2 (16.7)		14 (41.2)		
Single		15 (32.6)		6 (50.0)		9 (26.5)		
Widowed		4 (8.7)		2 (16.7)		2 (5.9)		
Education, years	16.18 (2.43)		16.25 (2.49)		16.15 (2.45)		.906	0.04
Annual household income, U.S.$	41,990.48 (25,751.92)		39,375.33 (27,385.25)		42,913.47 (25,513.97)		.687	0.134
Monthly SSA payment, U.S.$	1,446.24 (1,244.32)		1,627.25 (2,002.61)		1,382.35 (870.87)		.564	0.159
RAVLT LoT	17.28 (8.55)		15.92 (9.37)		17.81 (8.32)		.522	0.213
LFDRS total	13.85 (9.70)		8.25 (3.39)		15.82 (10.44)		.018	0.976

*Notes:* MCI = mild cognitive impairment; PCI = perceived cognitive impairment; CN = cognitively normal; SMD = standardized mean differences; SSA = Social Security Administration; RAVLT LoT = Rey Auditory Verbal Learning Test Learning over Trials; LFDRS = Lichtenberg Financial Decision Rating Scale; *SD* = standard deviation.

Other descriptive information was collected during the interviews. Twenty-three of the 46 participants experienced some wealth loss, and eight of the 23 who had experienced wealth loss lost less than 10% of their annual regular income. In contrast, 15 of the 46 participants lost more than 10% of their income. In five cases, we identified missed bills or late fees; 24 participants had an excessive payment in a single category; 26 were helping another person financially on a regular basis; and nine had been financially exploited. Five participants had been the victims of scams, and four had been exploited financially by family and/or friends. We were not able to determine the amount of money lost.

##### Overall associations.―

**Figure 1.**
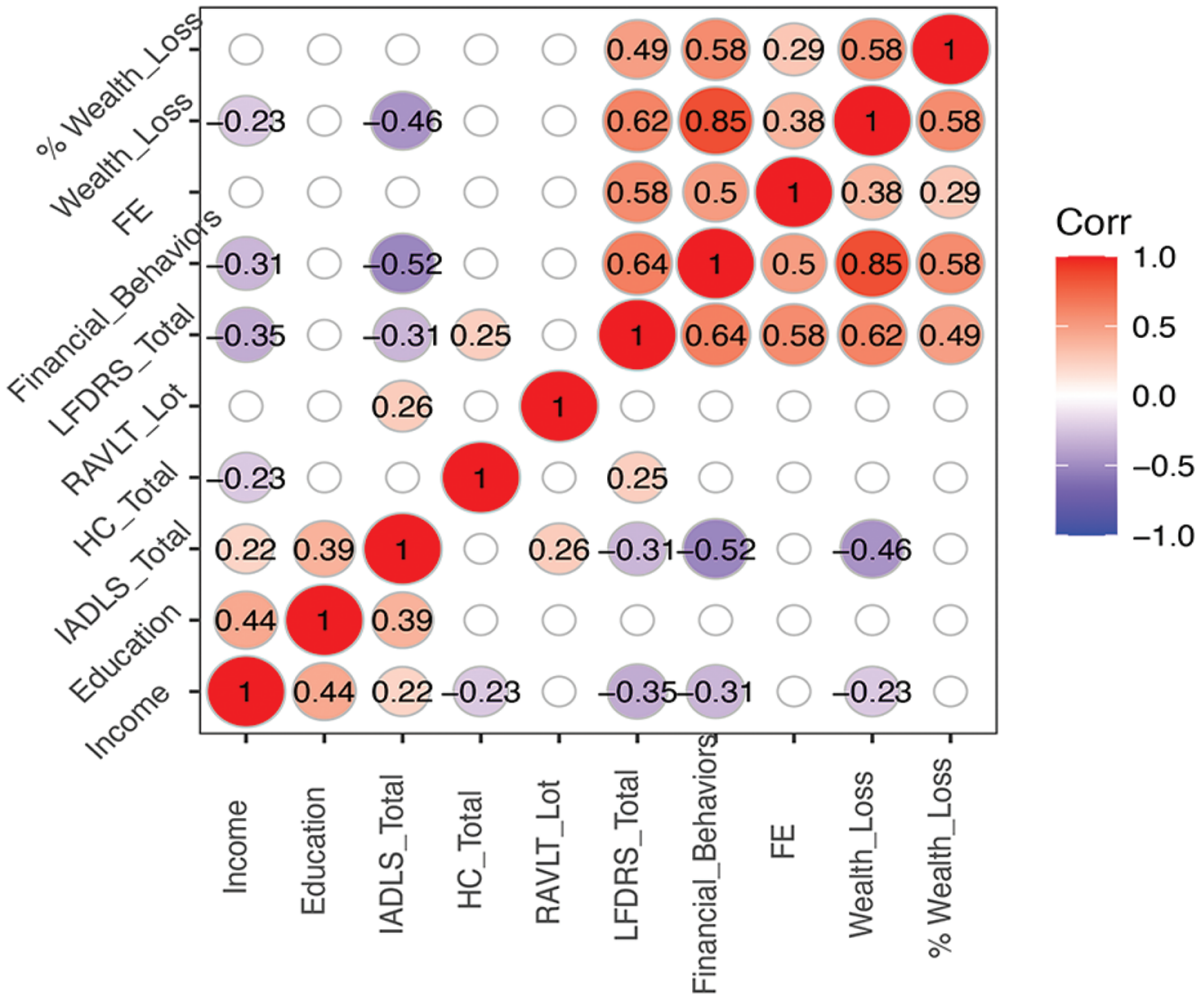
Correlation plot (*p* < .2 presented in plot).*Notes:* FE = financial exploitation; LFDRS = Lichtenberg Financial Decision Rating Scale; RAVLT LoT = Rey Auditory Verbal Learning Test Learning over Trial; HC = health conditions; IADLS = instrumental activities of daily living scale. Financial behavior index = count of the following: missed bills, late fees, regular financial assistance to others, and overpayment in one expenditure category. Corresponding correlation matrix values are presented in Supplementary Table 1.

The pairwise correlation plot for socioeconomic, health, cognitive function, financial decision-making, and financial behaviors and outcomes are presented in Figure 1. First, worse financial behaviors were linked to a higher likelihood of FE and wealth loss. Second, higher financial decision-making scores (LFDRS) were also consistently linked to worse financial behaviors and outcomes. Third, cognitive function, based on the RAVLT-LoT score, was not associated with either financial behaviors or outcomes. Finally, higher-income but not education was linked to a lower likelihood of engaging in poor financial behaviors (the financial behaviors index) as well as a lower likelihood of wealth loss.

##### Cognitive status and financial behaviors.―

**Figure 2.**
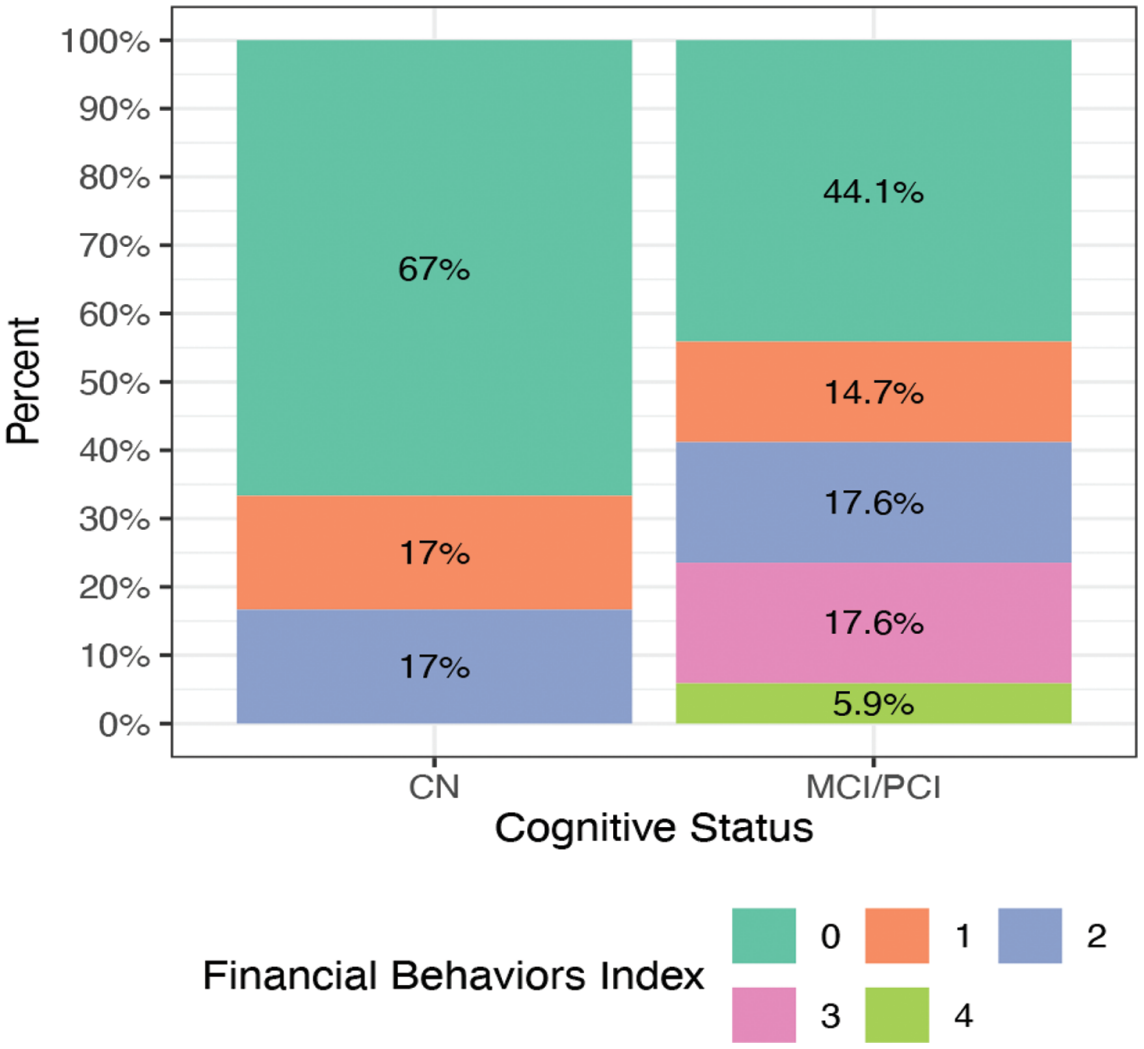
Prevalence for counts of problematic financial behaviors by cognitive status.*Notes:* CN = cognitively normal; MCI = mild cognitive impairment; PCI = perceived cognitive impairment.

The distribution of problematic financial behaviors by cognitive status is presented in Figure 2. Fifty-six percent of MCI/PCI individuals had evidence of problematic financial behaviors, compared with only 33% of CN participants. Of individuals with MCI/PCI. More importantly, close to one in four participants (23.5%) with MCI/PCI showed evidence of at least three problematic behaviors, whereas none of the CN participants did.

##### Adjusted associations (Table 2).―

In Model 1, which included adjustment for income, education, and cognitive status (i.e., MCI/PCI status), each unit increase in the count on the financial behavior index (indicating a higher number of problematic financial behaviors; range = 0–4) was associated with a 2.48 increase in the odds ratios of FE (OR = 2.48; 95% confidence interval [CI] = [1.21;5.09]; *p* = .013). The odds ratios for the financial behavior index were slightly attenuated (7.7% decrease in magnitude) by additional adjustment (Model 2) for cognitive function (RAVLT-LoT test) and IADL status (OR = 2.29; 95% CI = [1.04;5.01]; *p* = .039). Also, including the LFDRS in the model (Model 3) completely explained the association (OR = 1.89; *p* = .145) between the financial behaviors index and FE. In Model 3, each unit increase in LFDRS scores increased the odds ratios for FE (OR = 1.16; 95% CI = [1.01;1.34]; *p* = .042).

**Table 2.** Association Between Financial Behaviors, LFDRS, FEVS, and (1) Financial Exploitation and (2) Wealth Loss

Variable		Financial exploitation		% Wealth loss	
		OR	(95% CI) *p* value	*b*	(95% CI) *p* value
Model 1	Financial behaviors index	2.48	(1.21;5.09) *p* = .013	12.18	(5.89;18.46) *p* < .001
Model 2	Financial behaviors index	2.29	(1.04;5.01) *p* = .039	11.28	(5.49;17.06) *p* < .001
Model 3	Financial behaviors index	1.89	(0.8;4.47) *p* = .145	10.82	(3.15;18.49) *p* = .007
	LFDRS	1.16	(1.01;1.34) *p* = .042	0.15	(−1.21;1.51) *p* = .821

*Notes:* Financial behaviors index = count of the following: missed bills, late fees, regular financial assistance to others, and overpayment in one expenditure category. Model 1 adjusts for income, education, and MCI/PCI status. Model 2 additionally adjusts for cognitive function (Rey Auditory Verbal Learning Test Learning over Trial [RAVLT LoT]) and IADLs. Model 3 adds the LFDRS. LFDRS = Lichtenberg Financial Decision Rating Scale; OR = odds ratio; CI = confidence interval; FEVS = Financial Exploitation Vulnerability Survey; MCI = mild cognitive impairment; PCI = perceived cognitive impairment; IADL = instrumental activities of daily living.

Adjusting for income, education, and cognitive status (MCI/PCI status), each unit increase in the count on the financial behaviors index was associated with a (Model 1) higher percentage (12.18%) of wealth loss (*b* = 12.18; 95% CI = [5.89;18.46]; *p* < .001). The association between the financial behavior index and percentage wealth loss remained consistent in Model 2 (*b* = 11.28; 95% CI = [5.49;17.06]; *p* < .001) and Model 3 (*b* = 10.82; 95% CI = [3.15;18.49]; *p* = .007), which included additional adjustments for cognitive function (RAVLT LoT), IADLs, and the LFDRS. In Model 3, the LFDRS was not significantly linked to wealth loss (*b* = 0.15; *p* = .821).

## Discussion

Three important findings emerge from this feasibility study of wealth loss and FE during early memory loss. First, real-world personal finance analysis, achieved by examining an older person’s checking account statements, is feasible and meaningful. Through a structured set of analytic steps, we were able to review checking account statements prior to interviews, identify patterns in financial behaviors and outcomes, and collect important details during the interview with the older participant. By doing so, we were able to establish, with a high degree of certainty, whether there was evidence of wealth loss and/or FE for each older person in the study. The feasibility of a person-centered approach to real-world financial management, as proposed by the Institute of Medicine/National Academy of Sciences report, is supported by the results of this study. A person-centered approach to financial management has significant ramifications in terms of who may or may not need a conservator or representative payee and the detection of early signs of financial mismanagement, which can render an older adult more vulnerable to wealth loss and FE. Simply declaring that individuals with memory loss lack financial capacity is not supported by these data and runs counter to the principles of autonomy that are so deeply ingrained in our approach to decision-making and responsibility.

Third, early memory loss, financial decision-making, and financial management behaviors were linked in important ways that contribute to the risk of wealth loss and FE. For instance, financial decision-making scores that reflect higher risk or vulnerability were linked to wealth loss in those with early memory problems, yet financial outcomes were not related to scores on a standard test of learning and memory. This finding is consistent with the findings of Boyle et al. (2012) and Han et al. (2015), who found that while the constructs are associated, financial decision-making is a separate construct from cognitive functioning. The majority of our study participants had PCI, which means that they did not have objective evidence of decline but rated themselves as having some cognitive decline. Cognitive vulnerability, either perceived or objectively verified on cognitive testing, was related to an increased number of risky financial management behaviors, including excessive payments in one category, missed bills or late fees, and helping another financially on a regular basis.

Our study suggests important changes in checking account management and financial behavior. The financial capacity index (Marson, 2001), for example, contains tasks related to identifying cash, calculating cash transactions, bill payment, and checkbook management. Our study explored those domains by examining patterns of cash withdrawals and of writing checks for bill payments. We did not find strong evidence that late fees (only in 11% of participants) or cash withdrawal patterns were strong risk factors for wealth loss. Contrary to our expectations, most of the older people we examined did not use ATMs to withdraw cash, but frequently received extra cash when using a debit card to purchase products. Thus, cash withdrawal patterns were often obscured in the data and could not be used in our analyses. Second, most older adults interviewed wrote only three to five checks per month. Bills were largely paid online (often through automatic payments) by major credit cards that were paid monthly. The use of automatic bill payments reduced the risk of missed bills and can be viewed as a compensatory strategy. In addition, expenditures varied significantly across the calendar year due to expenses such as taxes or one-time purchases.

The study has several limitations. First, the sample size was modest, and tests were largely descriptive in nature. Second, after 11 in-person interviews with participants, all remaining work was conducted via telephone interviews and assessments due to COVID-19 restrictions. As a result, we were forced to shorten and streamline our battery for cognitive data collection (e.g., we eliminated our Trailmaking and Stroop executive functioning tests). Reviewing financial statements and conducting interviews to substantiate income values and expenditures is time-consuming and complicated. When we have a larger data set, we will investigate whether it is feasible to use simpler indices obtained simply by examining checking account statements.

Despite these limitations, the study’s strong conceptual and empirical basis contributes innovative approaches to the field. The findings from this feasibility study have significant implications for clinical practice because neurocognitive decline is well known to affect the domains of financial capacity (Marson, 2016). Approaches to assessing these domains have included hypothetical and objective measures of financial knowledge, management of checking and other accounts, and decision-making. Our work demonstrates an alternative approach and methods for assessing the financial management domain of financial capacity using person-centered principles, which we believe are critical in the presence of diminished or impaired cognition. Mast (2011) described a person-centered approach to the assessment of individuals experiencing neurocognitive disorders as combining important contextual features and standardized assessment methods. Contextual features were defined as those aspects of an individual’s life and history that affect their values, beliefs, and behaviors.

## Supplementary Material

igac038_suppl_Supplementary_Material

## References

[CIT0001] Angrisani, M., & Lee, J. (2018). Cognitive decline and household financial decisions at older ages. Journal of the Economics of Ageing, 13, 86–101. doi:10.1016/j.jeoa.2018.03.003 31572663 PMC6768425

[CIT0002] Appelbaum, P. S., & Grisso, T. (1988). Assessing patients’ capacities to consent to treatment. New England Journal of Medicine, 319, 1635–1638. doi:10.1056/NEJM198812223192504 3200278

[CIT0003] Boyle, P. A., Wilson, R. S., Yu, L. Y., Buchman, A. S., & Bennett, D. A. (2012). Poor decision making is a consequence of cognitive decline among older persons without Alzheimer’s disease or mild cognitive impairment. PLoS One, 7(8), 1–5. doi:10.1371/journal.pone.0043647 PMC3423371 22916287

[CIT0004] Campbell, R., Gross, E., & Lichtenberg, P. A. (2019). Cross-validation of the screening scale in an adult protective services sample. Journal of Elder Abuse and Neglect, 31(1), 25–37. doi: 10.1080/08946566.2018.1531098 30406729 PMC6424609

[CIT0005] Graf, C . (2008). The Lawton instrumental activities of daily living scale. American Journal of Nursing, 108(4), 52–62. doi:10.1097/01.naj.0000314810.46029.74 18367931

[CIT0006] Han, S. D., Boyle, P. A., James, B. D., Yu, L. Y., Barnes, L. L., & Bennett, D. A. (2015). Discrepancies between cognition and decision making in older adults. Aging—Clinical and Experimental Research, 28, 99–108. doi:10.1007/s40520-015-0375-7 25995167 PMC4654982

[CIT0007] Hsu, J. W., & Willis, R. (2013). Dementia risk and financial decision making by older households: The impact of information. Journal of Human Capital, 17, 340–337. http://www.journals.uchicago.edu/doi/pdfplus/10.1086/674105 10.2139/ssrn.2339225 PMC4267321 25525476

[CIT0008] Lezak, M . (1995) Neuropsychological assessment (3rd ed.). Oxford University Press.

[CIT0009] Lichtenberg, P. A., Ficker, L., Rahman-Filipiak, A., Tatro, R., Farrell, C., Speir, J. J., Mall, S. J., Simasko, P., Collens, H. H., & Jackman, J. D. Jr. (2016). The Lichtenberg Financial Decision Screening Scale (LFDSS): A new tool for assessing financial decision making and preventing financial exploitation. Journal of Elder Abuse and Neglect, 28(3), 134–151. doi:10.1080/08946566.2016.1168333 27010780 PMC4938730

[CIT0010] Lichtenberg, P. A., Gross, E., & Ficker, L. (2020). Quantifying risk of financial incapacity and financial exploitation in community-dwelling older adults: Utility of a scoring system for the Lichtenberg Financial Decision making Rating Scale. Clinical Gerontologist, 43, 266–280. doi:10.1080/07317115.2018.1485812 29883276 PMC6286690

[CIT0011] Lichtenberg, P. A., Ocepek-Welikson, K., Ficker, L. J., Gross, E., Rahman-Filipiak, A., & Teresi, J. (2017). Conceptual and empirical approaches to financial decision making in older adults: Results from a financial decision-making rating scale. Clinical Gerontologist, 41, 42–65. doi:10.1080/07317115.2017.1367748 29077531 PMC5766370

[CIT0012] Lichtenberg, P. A., Stoltman, J., Ficker, L. J., Iris, M., & Mast, B. T. (2015). A person-centered approach to financial capacity assessment: Preliminary development of a new rating scale. Clinical Gerontologist, 38, 49–67. doi:10.1080/07317115.2014.970318 25866438 PMC4392714

[CIT0013] Marson, D . (2016). Conceptual models and guidelines for clinical assessment of financial capacity. Archives of Clinical Neuropsychology, 31(6), 541–553. doi:10.1093/arclin/acw052 27506235 PMC5007080

[CIT0014] Marson, D. C . (2001). Loss of financial competency in dementia: Conceptual and empirical approaches. Aging, Neuropsychology, and Cognition, 8, 164–181. doi:10.1076/anec.8.3.164.827

[CIT0015] Mast, B.T . (2011). Whole person dementia assessment. Health Professions Press.

[CIT0016] Nicholas, L. H., Langa, K. M., Bynum, J. P., & Hsu, J. W. (2021). Financial presentation of Alzheimer’s disease and related dementia. JAMA Internal Medicine, 181, 220–227. doi:10.1001/jamainternmed.2020.6432 33252621 PMC7851732

[CIT0017] Rey, A. (1958). L’examen clinique en psychologie. Presses Universitaires de France.

[CIT0018] Stewart, C. C., Yu, L., Wilson, R. S., Bennett, D. A., & Boyle, P. A. (2019). Healthcare and financial decision making and incident adverse cognitive outcomes among older adults. Journal of the American Geriatrics Society, 67, 1590–1595. doi:10.1111/jgs.15880 30882910 PMC9801701

