# Supplementary material for: The WALLET Study: Examining Early Memory Loss and Personal Finance
Source: Innov Aging. 2022 May 26;6(5):igac038. doi: 10.1093/geroni/igac038 (PMC9250658; doi:10.1093/geroni/igac038)
Supplement: igac038_suppl_Supplementary_Material [file igac038_suppl_supplementary_material.docx]

**Supplementary Table 1.** **Correlation matrix.**

| **Variable** | **Income** | **Education** | **IADLS Total** | **HC Total** | **RAVLT Lot** | **LFDRS Total** | **FBI** | **FE** | **Wealth Loss** | **% Wealth Loss** |
| --- | --- | --- | --- | --- | --- | --- | --- | --- | --- | --- |
| Income | 1 |  |  |  |  |  |  |  |  |  |
| Education | 0.444 | 1 |  |  |  |  |  |  |  |  |
| IADLS Total | 0.22 | 0.39 | 1 |  |  |  |  |  |  |  |
| HC Total | -0.227 | 0.036 | -0.18 | 1 |  |  |  |  |  |  |
| RAVLT Lot | 0.155 | 0.038 | 0.261 | 0.009 | 1 |  |  |  |  |  |
| LFDRS Total | -0.352 | -0.04 | -0.308 | 0.253 | -0.11 | 1 |  |  |  |  |
| FBI | -0.31 | -0.122 | -0.522 | 0.112 | -0.169 | 0.64 | 1 |  |  |  |
| FE | -0.089 | 0.055 | -0.134 | -0.047 | 0.048 | 0.579 | 0.497 | 1 |  |  |
| Wealth Loss | -0.229 | -0.149 | -0.461 | 0.075 | -0.138 | 0.623 | 0.847 | 0.384 | 1 |  |
| % Wealth Loss | 0.031 | 0.1 | -0.133 | 0.099 | 0.106 | 0.487 | 0.577 | 0.29 | 0.584 | 1 |

*Notes*. IADLS = Instrumental Activities of Daily Living Scale; HC = Health Conditions; RAVLT = Rey Auditory Verbal Learning Test Learning Over Trial; LFDRS = Lichtenberg Financial Decision Rating Scale; FBI = Financial Behaviors Index; FE = financial exploitation.
